# Perceived Decrease in Workplace Security Since the Beginning of the COVID-19 Pandemic: The Importance of Management Styles and Work-Related Attitudes

**DOI:** 10.3389/fpsyg.2021.635973

**Published:** 2021-08-19

**Authors:** Anna Wojtkowska, Ernest Tyburski, Katarzyna Skalacka, Agata Gasiorowska

**Affiliations:** ^1^Institute of Psychology, SWPS University of Social Sciences and Humanities, Wroclaw, Poland; ^2^Institute of Psychology, University of Opole, Opole, Poland; ^3^Institute of Psychology, SWPS University of Social Sciences and Humanities, Poznan, Poland

**Keywords:** workplace security, psychological security, COVID-19, management style, satisfaction of work, psychological safety and trust

## Abstract

The coronavirus disease 2019 (COVID-19) pandemic has reduced the sense of security of people in everyday life. The efforts of managers in the workplace to minimize the health risks and economic damage, however, can provide the employees with a greater sense of security. The aim of this study was to identify the types of workplace responses to the pandemic outbreak with respect to the characteristics of employees and their employers accomplishing the differences in subjective sense of workplace security before the pandemic and during the outbreak. Three hundred and thirty-seven Polish employees completed an online survey during the first 2 weeks of the COVID-19 pandemic in March 2020. Using the cluster analysis, we identified four subgroups of employees differing in their sense of workplace security, work-related psychological factors, and perceived management styles of their supervisors. Employees led by developers and executive managers sustained a high sense of work security and positive attitude to work, while those led by compromisers and deserter managers suffered from the highest drop of subjective security. In this study, we proposed how employees can be protected from overreactions and unnecessary panic in a time of global crisis by virtue of the psychological competences of their supervisors and employers.

## Introduction

The ongoing coronavirus disease 2019 (COVID-19) pandemic caused by the new severe acute respiratory syndrome coronavirus 2 (SARS-CoV-2) is potentially the greatest threat to global health and security since World War II. Many managers and employees were taken by surprise by the sudden onset and rapid spread of the disease that forced the immediate implementation of changes to their daily habits, work organization, and social contacts. Although we faced previous pandemics (e.g., SARS in 2003, influenza A H1N1 in 2009, and Ebola in 2014), we have learned only a few lessons on how to cope with a crisis as fast-developing and as worldwide as this one. The COVID-19 pandemic has forced employers to deal with such issues as unplanned absences of a large pool of employees, customers leaving, and the need for protecting oneself and others in a diverse range of work environments. To protect employees and clients from this invisible threat, as well as to protect the company itself from the crisis and perturbations in the global economy, leaders must provide their employees with knowledge and skills that, in addition to personal dispositions, are the bases for successful adaptation to quickly changing and stressful circumstances (Berger et al., 2019).

The fact that the ongoing pandemic will cause—and has already caused—widespread changes to all spheres of human functioning is indisputable (Pfefferbaum and North, 2020; Zandifar and Badrfam, 2020). At the moment, we cannot be sure about the scope of these changes nor their long-term influence. However, the first published reports confirm early signs of a growing danger to mental health and global social conditions. Research reports a significant increase in stress, depression, and anxiety in many countries, including China (Wang et al., 2020), Italy (Amerio et al., 2020), Spain (Ozamiz-Etxebarria et al., 2020), Germany (Bauerle et al., 2020), France (Husky et al., 2020), the United Kingdom (Smith et al., 2020) and the variety of other populations observed during COVID-19 and previous epidemics (Torales et al., 2020). For example, the first Chinese large-scale research showed that even more than 50–70% of citizens were suffering moderate or high intensities of serious psychological symptoms, such as depression, anxiety, phobic anxiety (Wang et al., 2020), obsessive compulsive symptoms, interpersonal sensitivity, and other stress-related mental issues (Tian et al., 2020). Beside serious mental problems, the ongoing pandemic also increased the levels of xenophobia, stigma, and racism (Jakovljevic et al., 2020). An emotional dysregulation increasing the psychological distress was observed not only in the general population (Moccia et al., 2020; Janiri et al., 2021) but also in clinical samples of people with affective (Di Nicola et al., 2020) and neurological disorders (Taquet et al., 2021). It seems that people with poorer general health, women, students, young employees, individuals aged older than 50 years, and medical staff might be particularly sensitive to the psychological difficulties of the crisis (Tian et al., 2020; Wang et al., 2020). Indeed, those initially most exposed to direct danger—medical workers from hospitals in Wuhan—have demonstrated increased scores for depression, exhaustion, and anxiety. The highest psychological cost was paid by nurses (Lai et al., 2020). A suffering of healthcare workers has been already observed in China (Pappa et al., 2020), India (Chew et al., 2020), the United States (Shechter et al., 2020), Spain (García-Fernández et al., 2007), and other countries.

The first longitudinal studies also confirmed the persistent deterioration of mental health along with the development of the pandemic (Daly et al., 2020; Niedzwiedz et al., 2020; O'Connor et al., 2020). While there is already convincing evidence of the impact of the COVID-19 pandemic on the mental health of healthcare workers, the information of such effects on the general population of adults working in a variety of industries and work environments is still relatively scarce. To understand possible long-term costs paid by employees of industries other than medical, it might be useful to infer from other crises that have shattered global safety. Some conclusions can be drawn from the reaction of the world to the terrorist attack on the World Trade Center (WTC) in 2001, which, similarly to the pandemic, shook the global sense of security and made people aware of a previously underestimated threat. The effects of posttraumatic stress among the employees of the different professions directly engaged in the WTC rescue and reconstruction have been observed over both the short and long terms. For policemen, posttraumatic stress disorder (PTSD) appeared even 10–11 years after the tragedy, and being unemployed, unable to work due to health issues, or being retired was significantly associated with the comorbidity of mental health (Bowler et al., 2016). Among the multicultural population of citizens who survived the attack, keeping their jobs was one of the factors protecting them from both chronic and late-onset PTSD (Kung et al., 2018). Cukor et al. (2011) observed that subjective perception of a lack of security was the best predictor of probable PTSD also for American employees who were not involved in rescue after the WTC attack.

Direct actions of managers, such as providing the personal protective equipment, flexibility of work schedules, and ensuring stability of employment, can physically protect employees from global threats and the psychological effects thereof. After having dealt with the second wave of the 2009 A/H1N1 flu pandemic, the willingness of nurses to work in the next possible flu epidemic was greatly determined not only by the availability of the personal protective equipment but also by having emotional and informational support (Hartley and Cabanac, 2013). A qualitative research on willingness to work during influenza epidemics among healthcare workers indicated that the behaviors undertaken by managers were crucial for decisions of nurses related to readiness to work. Information flow and work guidance influenced individual perceptions of personal risk in the workplace, which, in turn, influenced personal choices concerning whether to work or not during that epidemic (Ives et al., 2009). Evidence from previous epidemics indicated that informational support could play a central role in human resource management in times of crisis. The open sharing of information about the situation and objective danger can make employees feel that they are important for the institution and have a role to play in coping with crisis situations, which in turn may affect the sense of security of employees in the workplace and can help in avoiding burn-out syndrome (Gutierrez et al., 2013). In Florida, medical workers who had read the H1N1 influenza pandemic plan were much more ready to work in further epidemiological danger than those who did not read it (Basta et al., 2009). Hence, while a rapidly developing pandemic stays mostly out of control, leaders have some influence on the psychological response of employees to this threat. The constructive management style may decrease negative emotions and hence support the health and well-being of employees (Restubog et al., 2020). However, there still is a knowledge gap concerning how to manage in a crisis such as pandemic and how to prepare managers to manage in such conditions (Hirpara and Taylor, 2020). Current lessons from management during the COVID-19 outbreak revealed that leaders in epidemiological crises should especially communicate with employees frequently and strive to build trust in their teams.

According to the study by Kalina (2020), the most crucial competences for successful managing in a difficult time is to stay calm, stable, constantly educated, focused, trustworthy, visible, decisive, assertive, confident, flexible, open-minded, adaptive, authentic, honest, clear, creative, realistic, and credible. However, there is virtually no research on specific management styles that could be especially effective in coping with pandemic crises in a variety of work environments and industries other than healthcare. In this research, we focused on the classic three-dimensional (3D) management style theory that can be helpful in planning a management strategy for a variety of employees (Reddin, 1990; see also Reddin, 1967). This theory identifies eight main leadership styles and indicates that their effectiveness depends on specific requirements or circumstances under which the leader and his subordinates work, representing a situational approach to management and assuming that leadership styles are not objectively positive or negative (Fiedler, 1976). However, independent of the situational background, Reddin (1990) suggested that some leadership styles—such as bureaucrat, developer, benevolent autocrat, and executive—might be, in most cases, considered as more constructive than others, including a deserter, missionary, autocrat, and compromiser.

A manager representing the deserter style is isolated and often lacks interest both in tasks and in relationships. The missionary style, the second management style considered as less effective, is more relationship-focused than other styles, which can lead employees to overstate satisfaction but reduces productivity, especially in difficult and risky tasks. This style is mostly ineffective, as such managers prefer to be seen as a good person rather than an effective leader. In turn, the priority of an autocrat is the immediate success of the task over relationships and other considerations. Such an attitude often leads to too much pressure, thus lowering employee motivation and generating non-constructive emotions, such as lack of trust in authority and aversion to the leader. Finally, leaders who compromise identify the benefits of both task and relational orientation but are usually unable to make decisions confidently, which in turn leads to their ambiguous workplace behavior depending on the current situation and short-term requirements. Such leaders prefer to minimize the current problems rather than to maximize the long-term benefits and concentrate on employees who are helpful for their career or well-being, while being ready to sacrifice those who are not only more competent but also more demanding (Reddin, 1990).

Among the more effective leadership styles, Reddin (1990) distinguished a bureaucrat, who is really not interested in the success of tasks or relationships but simply follows all formal rules and norms. Such an approach can be beneficial, especially in large hierarchical companies and government institutions. In turn, a developer is focused on good relations with employees and also on high efficiency and considers a positive atmosphere in the workplace to be an important factor in the work efficiency and skills development. Contrary to the less-effective missionary style, having a developer as a supervisor requires a lot of commitment on the part of employees, but such a person also gives a lot of themselves. Then, benevolent autocrats focus on efficiency and, to a lesser extent, on good relations with employees. While they also make high demands on employees, they do not create a tense atmosphere in the workplace; hence, they differ from “ordinary” autocrats described earlier. Finally, an executive manager is task-, people-, and efficiency-oriented. A manager representing such a style sets ambitious goals for employees, has high personal results, maintains a positive atmosphere, is open to new ideas, and involves employees in the planning process (Reddin, 1967, 1990).

Applying different leadership styles can be useful for dealing with diverse types of problems, but, as we have already mentioned, some of them are generally more successful than others in daily management, as well as during times of crisis. In a correlation study involving executives, Limbare (2012) found that executives mostly preferred missionary leadership and appeasement conflict management, while deserter leadership and resignation conflict management were the most rejected (Limbare, 2012). Karim (2016) explored the model of *charismatic leadership* in a time of crisis and concluded that this framework is essential to assess the readiness of managers to deal with extreme events and often unavoidable difficulties. In comparison with the paradigm of Reddin, the charismatic leadership style combines the self-confidence typical of the executive style, the cooperation and flexibility characteristic of the developer style, and the empathy of the missionary leadership style. Other features of a charismatic manager include high motivation, empowerment, clarity of vision, caring about others, providing clear and simple instructions, coordination, loyalty, flexibility, poise, control, decision-making, the ability to command without hesitation, and readiness to accept leadership responsibility. These abilities are a predictive criterion for assessing whether a leader has the appropriate predisposition and qualifications for leadership in times of crisis (Karim, 2016).

Based on a review of the current knowledge, we assumed that the efforts that managers put into their workplaces in order to minimize health risks and economic damage might contribute to the successful implementation of protective measures and provide employees with a greater sense of security to decrease the psychological burden. Recent research has focused mainly on healthcare workers, but it seems that similar effects can also be observed in other professions and industries. For addressing this gap, it becomes crucial to look for psychological factors linked to better coping with ongoing crises in order to reduce short- and long-term damages from individual, local, and global perspectives and to provide informative suggestions for further research and coping strategies. Hence, the aim of this study was to investigate the types of workplace responses to the pandemic outbreak with respect to the characteristics of employees and their employers, accomplishing the differences in subjective sense of workplace security before the pandemic and during the outbreak. Using the cluster analysis, we aimed at identifying subgroups of employees differing in their sense of workplace security, work-related psychological factors, and perceived management styles of their supervisors.

## Materials and Methods

### Participants

We recruited 337 Polish employees (i.e., 273 women, 63 men, and 1 non-binary person) aged from 18 to 61 (*M*_age_ = 29.64, standard deviation [*SD*] = 8.22) with professional work experience from ~1 month to 37 years (*M* = 7.03 years, *SD* = 7.23). Most of the participants (64.1%, *n* = 216) were working in Poland, and 35.9% (*n* = 121) were working abroad, mostly in Germany (*n* = 45), the Netherlands (*n* = 10), Malta (*n* = 9), Spain (*n* = 8), England (*n* = 7), and France (*n* = 7). The detailed sociodemographic characteristics of the studied participants are presented in Table 1.

**Table 1:** Demographic and work-related characteristics of the study sample (*n* = 337).

**Variables**	***N* (*%*)**
**Education**	
Primary/secondary	23 (6.9%)
High school-general	78 (23.1%)
High school-technical	51 (15.1%)
Bachelor's degree	162 (48.1%)
Master's degree	23 (6.8%)
**Place of residence**	
Rural	22 (6.5%)
Small city	48 (14.2%)
Medium city	25 (7.4%)
Big city	56 (16.6%)
Very big city	186 (55.3%)
**Place of work**	
Rural	12 (3.6%)
Small city (<50,000 citizens)	48 (14.2%)
Medium city (50,000–150,000 citizens)	23 (6.8%)
Big city (150,000–500,000 citizens)	59 (17.5%)
Very big city (> 500,000 citizens)	195 (57.9%)
**Company size**	
Micro (<10 employees)	49 (14.5%)
Small (<49 employees)	81 (24.0%)
Medium (<250 employees)	70 (20.8%)
Big national (> 250)	39 (11.6%)
Big international (> 250)	98 (29.1%)
**Work position**	
Physical worker	48 (14.2%)
Executive worker	109 (32.3%)
Specialist	33 (9.8%)
Independent specialist	108 (32.0%)
Manager	32 (9.5%)
Others	7 (2.2%)

### Procedure

We collected the data using an online survey distributed *via* Facebook from March 13 to March 23, 2020, i.e., during the earlier days of the COVID-19 pandemic in Europe. After providing informed consent, participants were asked to fill in the survey organized into four sections. In the first section, we collected information about basic sociodemographic characteristics of the employees, employers, and companies, such as gender, age, level of education, place of residence and work, seniority, company size, and industry. The second section contained questions about the work-related factors describing the subjective sense of engagement of employees in work (“How do you assess your general engagement in your current job?”), general work satisfaction (“How do you assess your general work satisfaction?”), work atmosphere satisfaction (“How do you assess your satisfaction with the atmosphere at work?”), and feelings of trust in the manager (“How do you assess your general trust in your manager?”). Participants answered these questions using a scale from 1 = “very low” to 7 = “very high.” Further two sections measured manager acceptance (“How do you assess your agreement with the sentence ‘I would not change anything about my manager'?”) and willingness to change the current job (“Do you wish to change your job in the near future?”) and were answered using a scale from 1 = “strongly disagree” to 7 = “strongly agree.”

The third section of the survey contained two assessments of the subjective sense of workplace security of participants. Using a scale from 1 = “very low” to 7 = “very high,” the participants assessed their present sense of security (“How do you assess your workplace security right now?”) and retrospectively assessed their security before the COVID-19 pandemic outbreak (“How do you assess your workplace security before the COVID-19 pandemic?”). The final part of the survey contained the questionnaire by Reddin based on the 3D management style theory (Reddin, 1967, 1990, 2005) that we used to assess the perception of leadership styles of employees expressed by their managers. This questionnaire consists of eight questions with eight answers each, and participants evaluated the basic managerial competences of their supervisors by choosing one of the characteristics representing eight leadership styles described in the theoretical introduction (Cronbach's α 0.28–0.60). Therefore, each leadership style can be scored from 0 to 8 points, and the sum for all styles should be always 8.

### Analytical Approach

We conducted a statistical analysis using the IBM SPSS Statistics 25 software. We performed a two-step cluster analysis with iterative partitioning methods in line with guidelines provided by Clatworthy et al. (2010). We used psychological variables related to work and work security, including (1) subjective sense of workplace security before and during the pandemic outbreak, (2) eight management styles, and (3) six work-related factors, namely, work engagement, general work satisfaction, atmosphere satisfaction, trust in the manager, manager acceptance, and willingness to change the current job. All variables were *Z*-scored before analyses. We verified the similarity between variables included in the cluster analysis using Pearson's correlation, and we found weak to moderate correlations confirming that they were related but not equivalent (Table 2).

**Table 2:** Descriptive statistics and correlations between variables measured in the study.

	***M***	***SD***	**1**	**2**	**3**	**4**	**5**	**6**	**7**	**8**	**9**	**10**	**11**	**12**	**13**	**14**	**15**	**16**	**17**	**18**	**19**	**20**	**21**	**22**
**Subjective workplace security**																								
1. Before the pandemic	5.80	1.48	-	-	-	-	-	-	-	-	-	-	-	-	-	-	-	-	-	-	-	-	-	-
2. At the beginning of the pandemic	3.81	2.16	0.58^***^	-	-	-	-	-	-	-	-	-	-	-	-	-	-	-	-	-	-	-	-	-
3. Perceived change	−1.99	1.81	−0.02	0.74^***^	-	-	-	-	-	-	-	-	-	-	-	-	-	-	-	-	-	-	-	-
**Leadership styles:**																								
4. Deserter	0.47	0.80	−0.26^***^	−0.30^***^	−0.20^***^	-	-	-	-	-	-	-	-	-	-	-	-	-	-	-	-	-	-	-
5. Missionary	0.69	0.90	0.07	0.13^*^	0.15^**^	−0.11^*^	-	-	-	-	-	-	-	-	-	-	-	-	-	-	-	-	-	-
6. Autocrat	1.13	1.25	−0.31^***^	−0.40^***^	−0.24^***^	0.21^***^	−0.14^**^	-	-	-	-	-	-	-	-	-	-	-	-	-	-	-	-	-
7. Compromiser	1.36	1.54	−0.23^***^	−0.32^***^	−0.27^***^	0.33^***^	−0.04	0.24^***^	-	-	-	-	-	-	-	-	-	-	-	-	-	-	-	-
8. Bureaucrat	0.79	0.98	−0.15^**^	−0.14^*^	−0.09	0.07	−0.22^***^	0.13^*^	0.05	-	-	-	-	-	-	-	-	-	-	-	-	-	-	-
9. Developer	1.11	1.30	0.39^***^	0.43^***^	0.24^***^	−0.33^***^	0.11^*^	−0.39^***^	−0.44^***^	−0.22^***^	-	-	-	-	-	-	-	-	-	-	-	-	-	-
10. Benevolent	1.12	1.14	0.08	0.16^**^	0.12^*^	−0.25^***^	−0.05	−0.28^***^	−0.41^***^	−0.08	0.27^***^	-	-	-	-	-	-	-	-	-	-	-	-	-
11. Executive	1.00	1.24	0.33^***^	0.41^***^	0.25^***^	−0.42^***^	0.02	−0.38^***^	−0.49^***^	−0.14^*^	0.35^***^	0.30^***^	-	-	-	-	-	-	-	-	-	-	-	-
**Subjective sense of:**																								
12. Work engagement	5.96	1.06	0.13^**^	0.03	0.02	−0.11^*^	0.05	−0.07	−0.14^*^	−0.03	−0.10	0.01	0.12^*^	-	-	-	-	-	-	-	-	-	-	-
13. Global work satisfaction	4.88	1.55	0.41^***^	0.51^***^	0.34^***^	−0.25^***^	0.16	−0.34^***^	−0.34^***^	−0.16^**^	0.34^***^	0.15^**^	0.39^***^	0.38^***^	-	-	-	-	-	-	-	-	-	-
14. Atmosphere satisfaction	5.15	1.64	0.46^***^	0.46^***^	0.27^***^	−0.17^**^	0.17	−0.39^***^	−0.29^***^	−0.16^**^	0.43^***^	0.14^**^	0.30^***^	0.18^**^	0.65^***^	-	-	-	-	-	-	-	-	-
15. Trust in manager	4.61	1.88	0.46^***^	0.62^***^	0.44^***^	−0.39^***^	0.20	−0.52^***^	−0.41^***^	−0.19^**^	0.55^***^	0.30^***^	0.46^***^	0.19^***^	0.64^***^	0.62^***^	-	-	-	-	-	-	-	-
16. Manager acceptance	3.70	2.12	0.44^***^	0.55^***^	0.36^***^	−0.36^***^	0.16^**^	−0.50^***^	−0.40^***^	−0.13^*^	0.52^***^	0.44^***^	0.44^***^	0.17^**^	0.55^***^	0.58^***^	0.82^***^	-	-	-	-	-	-	-
17. Willingness to change work	3.92	2.25	−0.37^***^	−0.47^***^	−0.32^***^	0.26^***^	−0.11	0.39^***^	0.37^***^	0.16^**^	−0.39^***^	−0.35^***^	−0.35^***^	−0.18^**^	−0.66^***^	−0.59^***^	−0.59^***^	−0.56^***^	-	-	-		-	-
**Demographics**																								
18. Employee's age	29.64	8.22	0.03	0.09	0.12^*^	−0.01	0.06	0.06	−0.11	−0.05	−0.01	−0.01	0.12	0.18^**^	0.19^**^	−0.01	0.12^*^	0.11	−0.04	-	-	-	-	-
19. Seniority	7.03	7.23	−0.01	−0.08	0.13^*^	−0.00	0.04	−0.03	−0.12^*^	−0.08	0.02	0.00	0.10	0.15^**^	0.21^**^	0.01	0.16^**^	0.16^**^	−0.08	0.71^***^	-	-	-	-
20. Place of residence	4.88	1.58	0.10	0.06	−0.04	−0.01	−0.02	−0.06	0.05	0.04	0.05	0.04	−0.01	−0.11	−0.05	0.05	0.02	0.03	0.02	−0.15^**^	−0.12^*^	-	-	-
21. Place of work	5.04	1.42	−0.07	−0.01	−0.12^*^	0.03	0.01	0.01	0.00	0.01	0.00	0.03	0.01	−0.15	−0.04	0.02	−0.03	−0.01	0.06	−0.15	−0.09	0.77	-	-
22. Education	4.24	1.12	0.22^***^	0.22^***^	0.07	−0.05	−0.05	−0.09	−0.01	0.01	0.15^**^	0.05	0.08	−0.01	0.01	−0.02	0.07	0.06	0.01	0.21^***^	0.06	0.21^***^	0.13^*^	-
23. Company size	3.16	1.44	0.12^*^	0.10	0.05	−0.14^*^	−0.06	−0.07	−0.17^**^	0.11^*^	0.19^*^	0.05	0.14^*^	−0.02	0.02	0.01	0.11	0.13^*^	−0.04	0.07	0.04	0.01	0.02	0.08

* *p < 0.05*,

** *p < 0.01*,

*** *p < 0.001*.

We determined the number of clusters using the silhouette measure of cohesion and separation (Kaufman, and Rousseeuw, 1990). A two-step clustering suggested the existence of two to four independent clusters in our data. We chose a four-cluster solution; the two- and three-cluster solutions appeared to be trivial. Subsequently, we conducted a K-mean (non-hierarchical) cluster analysis. In such an analysis, the number of clusters is determined before the analysis, and the algorithms find the cluster center and assign the objects to the nearest cluster center. K-mean algorithms usually prefer clusters of approximately similar size (Vogt and Nagel, 1992), which we considered an advantage of this method in our study. The value of silhouette coefficient of cohesion and separation was around 0.2, so it indicated well the stability of four-cluster solutions to best explain the types of reactions exhibited by Polish employees and managers. To verify the validity of the clusters, we repeated the calculations on a subsample randomly selected from a total study group (Clatworthy et al., 2010). Even on a sample that was twice as small, the cluster arrangement was analogous, with the silhouette value dropped to lower than 0.1. Nineteen participants did not fit into any cluster and were excluded from further analyses.

## Results

### Descriptive Statistics

**Figure 1:**
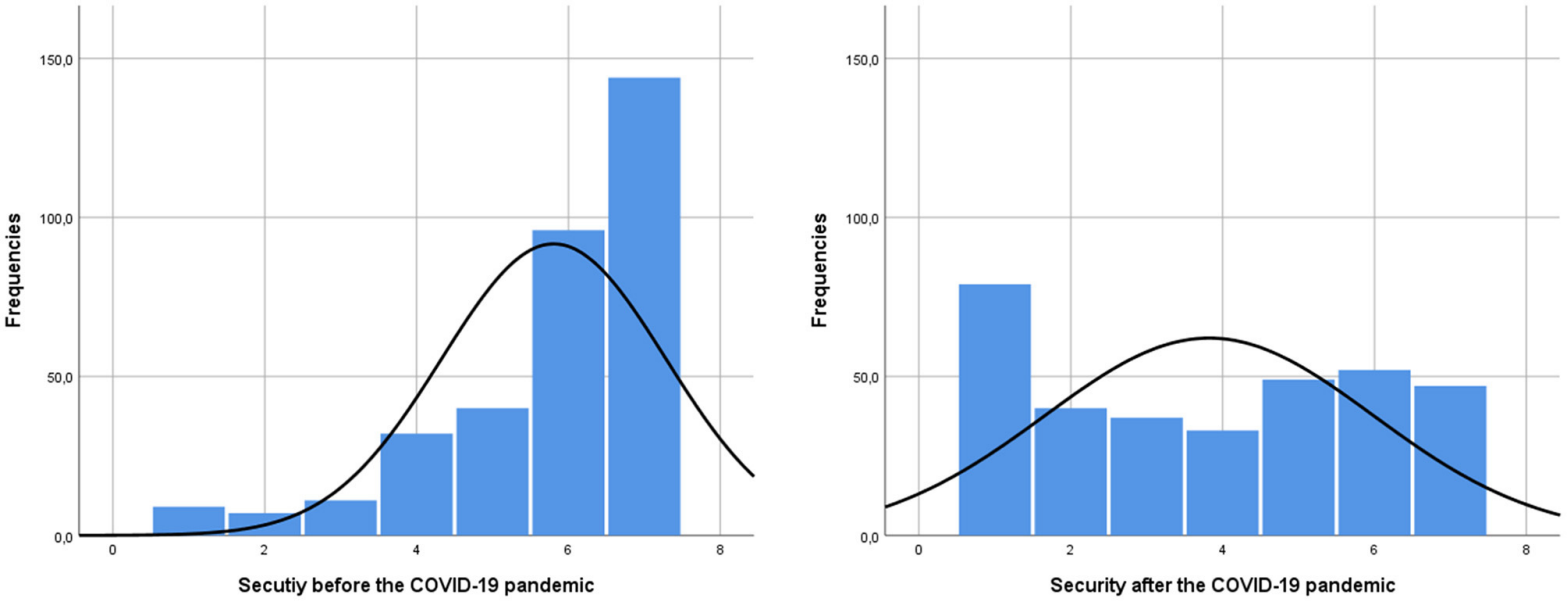
The distribution of subjective sense of workplace security before and after the pandemic outbreak.

The distribution of sense of security before and after the pandemic changed from left-skewed to more symmetric (Figure 1). Average retrospective-perceived safety before the pandemic was much higher (*M* = 5.81, *SD* = 1.48) than at the beginning of the pandemic (*M* = 3.82, *SD* = 2.17), *Z* = −13.48; *p* < 0.001. A quarter of the group reported that they did not experience any changes (25.0%, *n* = 86); 1.8% of the participants (*n* = 6) experienced an increase in their sense of security by 1–3 points, but most of the group reported a drop in their sense of security by 1–6 points.

While there were no differences in the sense of security before and after the pandemic between Poles working in Poland and abroad (*p*'s > 0.05), the former group reported a higher drop in their sense of security (*M* = −2.15, *SD* = 1.77) than the latter (*M* = −1.70, *SD* = 1.86), *Z* = 2.59, *p* = 0.010. We did not find gender differences with respect to the sense of security after the outbreak or with respect to the perceived change in the sense of security, while women scored higher on workplace security before COVID-19 (*M* = 5.90, *SD* = 1.41) than men (*M* = 5.40, *SD* = 1.69), *Z* = −2.401; *p* = 0.016. The age, seniority, and places of residence and work of an employee did not correlate with subjective security before and during the COVID-19 outbreak, but the higher levels of education were associated with a slightly higher subjective workplace security before and during the outbreak. We also found a very weak but significant correlation between security before the pandemic and company size. The descriptive statistics and correlations between variables are presented in Table 2.

### Cluster Analysis

**Figure 2:**
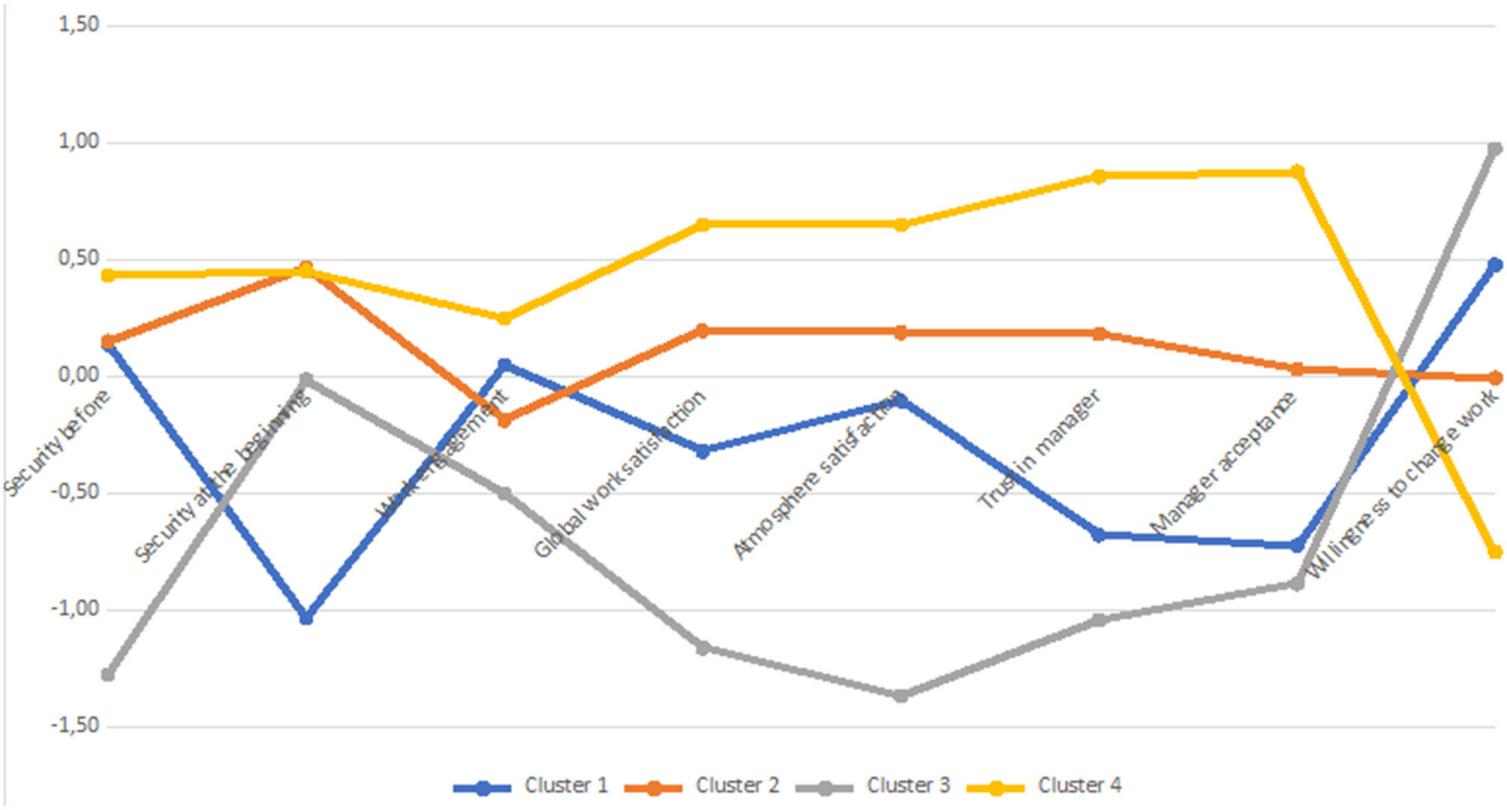
Characteristics of clusters in terms of subjective security and work-related variables.

**Figure 3:**
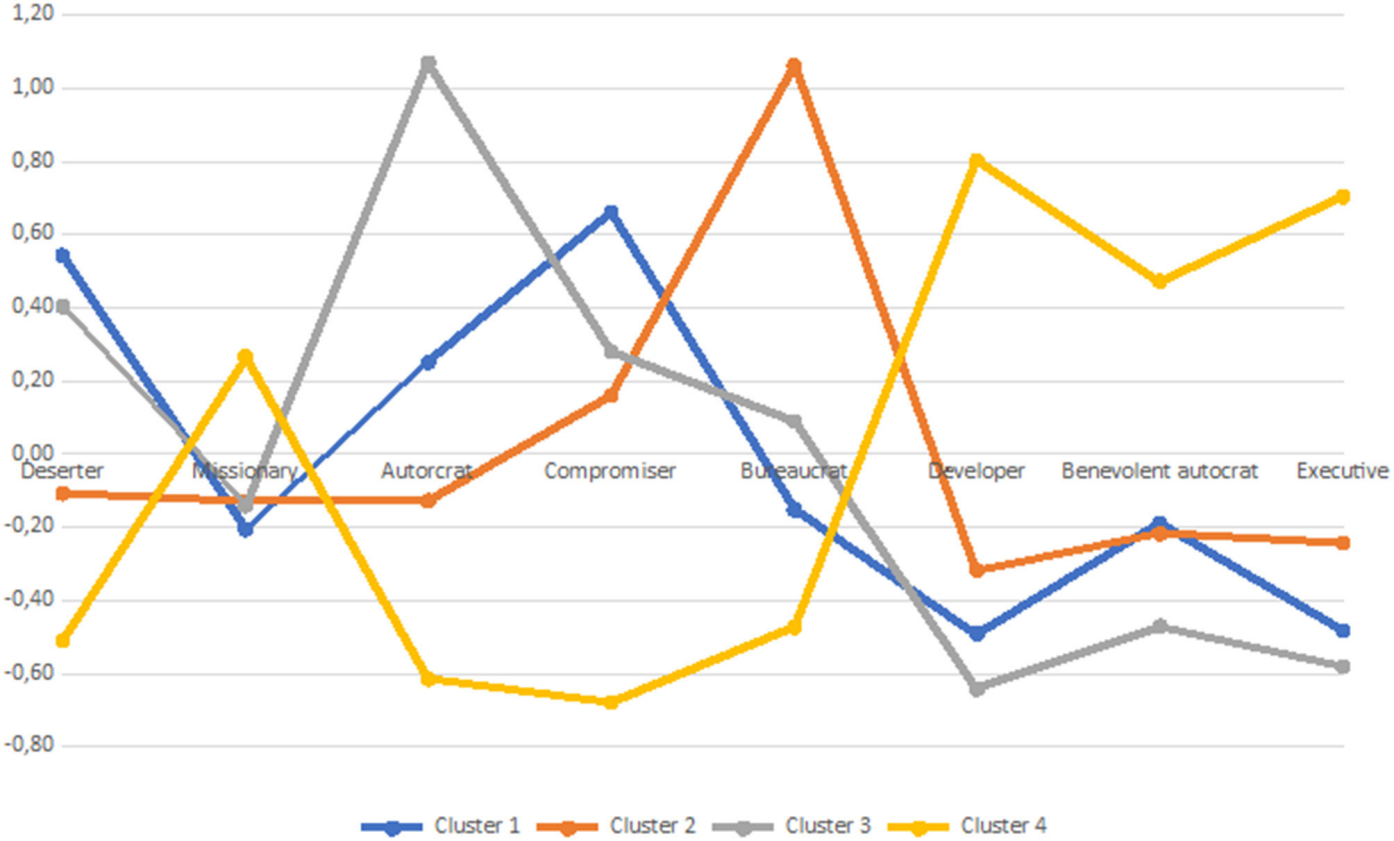
Characteristics of clusters in terms of perceived management styles.

The detailed characteristics of four clusters are presented in Table 3 and in Figures 2, 3. We found significant differences between clusters with respect to all the variables that were used in the cluster analysis (see Table 2), while we did not find significant differences between the clusters in terms of gender, χ^2^(3, n = 318) = 3.67, *p* = 0.299, age, *H* = −0.62, *p* = 0.534, seniority, *H* = −0.62, *p* = 0.535, education, χ^2^(12, n = 318) = 20.64, *p* = 0.056, place of residence, χ^2^(12, n = 318) = 13.10, *p* = 0.362, place of work, χ^2^(12, n = 318) = 4.50, *p* = 0.982, and company size, χ^2^(12, n = 318) = 11.71, *p* = 0.469. Although clusters differed with respect to subjective sense of workplace security before and during the pandemic outbreak, the perceived management styles of supervisors and the abovementioned six work-related factors did not differ with respect to any of the sociodemographic variables that we measured in this study. In the following part of this section, we provided a description of the clusters that were found in the collected data.

**Table 3:** Characteristics of clusters.

**Variables**	**Clusters**				**Cluster comparison**
	**1**	**2**	**3**	**4**	***H(3*)**
**Subjective workplace security**					
Before the pandemic	6.45 (1.03)^b^	6.05 (1.12)^a,b^	3.93 (1.58)	6.01 (1.04)^a^	110.84^***^
At the beginning of the pandemic	5.29 (1.69)^a^	4.90 (1.58)^a^	1.93 (1.22)^b^	2.16 (1.31)^b^	160.74^***^
Perceived change	−1.15 (1.34)^a^	−1.15 (1.60)^a^	−2.00 (1.41)	−3.85 (1.39)	119.20^***^
**Leadership styles**					
Deserter	0.08 (0.31)	0.40 (0.66)	0.83 (0.89)^a^	0.94 (1.04)^a^	70.66^***^
Missionary	0.98 (1.06)^c,e^	0.60 (0.82)^a,b,c^	0.60 (0.70)^b,d,e^	0.54 (0.81)^a,d^	12.36^***^
Autocrat	0.40 (0.63)	1.02 (0.99)^a^	2.51 (1.40)	1.49 (1.13)^a^	111.90^***^
Compromiser	0.36 (0.75)	1.68 (1.18)^a,b^	1.86 (1.52)^b,c^	2.45 (1.71)^a,c^	119.02^***^
Bureaucrat	0.36 (0.59)	1.84 (1.18)	0.91 (0.89)^a^	0.68 (0.77)^a^	84.28^***^
Developer	2.18 (1.41)	0.77 (0.74)^a^	0.32 (0.60)^b^	0.51 (0.80)^a,b^	128.58^***^
Benevolent autocrat	1.70 (1.12)	0.94 (1.04)^a,b^	0.63 (0.79)^b,c^	0.95 (1.18)^a,c^	47.20^***^
Executive	1.93 (1.34)	0.73 (0.96)^a,b^	0.32 (0.57)^b,c^	0.44 (0.71)^a,c^	110.50^***^
**Subjective sense of:**					
Work engagement	6.28 (0.82)^d^	5.74 (0.90)^a,b^	5.46 (1.51)^a,c^	6.04 (0.91)^b,c,d^	21.81^***^
Global work satisfaction	5.93 (1.01)	5.19 (0.85)	3.11 (1.33)	4.41 (1.38)	135.56^***^
Atmosphere satisfaction	6.22 (0.88)	5.50 (1.22)^a^	2.95 (1.37)	5.00 (1.17)^a^	145.09^***^
Trust in manager	6.24 (0.90)	4.97 (1.89)	2.67 (1.47)^a^	3.35 (1.36)^a^	196.08^***^
Manager acceptance	5.59 (1.35)	3.79 (1.60)	1.84 (1.33)^a^	2.19 (1.37)^a^	178.79^***^
Willingness to change work	2.23 (1.59)	3.89 (1.97)	6.11 (1.32)	4.99 (1.82)	138.59^***^

*Raw scores with standard deviations in parentheses. Means with the same superscript do not differ at p = 0.05*.

*** *p < 0.001*.

Cluster 1, consisting of *n* = 119 participants, was characterized by the highest level of the sense of security before the pandemic and the relatively smallest drop during the outbreak so that the subjective sense of workplace security in this cluster after the outbreak was the highest among all clusters. Participants in this cluster declared the highest level of work engagement, atmosphere and global work satisfaction, and the lowest level of turnover intention. They trusted their managers and accepted those managers to the highest extent among all four clusters. They perceived their managers as mainly demonstrating developer and executive styles of management, and also, to a lesser extent, they saw them as benevolent autocrats or missionaries. At the same time, they declared that their supervisors did not present such non-constructive styles as a compromiser, deserter, or autocrat.

Cluster 2, consisting of *n* = 80 participants, was similar to cluster 1 in terms of subjective sense of security before and after the outbreak of the COVID-19 pandemic. However, their work engagement, work satisfaction, and trust in the manager were lower than in the previous cluster, while their willingness to change job was significantly higher. They saw their managers not only as strongly bureaucratic and compromising, but also—to some extent—as autocratic or benevolently autocratic. Yet, they believed that their managers did not demonstrate deserter or missionary styles, so they did not avoid concentration on tasks, but they seemed to introduce strong norms and rules in order to minimize current problems rather than maximize the long-term benefits.

Participants from the following two clusters seemed to face difficulties despite pandemic. Participants from cluster 4 (*n* = 57) declared the lowest level of sense of security following the outbreak of the pandemic, and they did not report the highest drop in this variable only because they indicated it was already low before the pandemic. They saw their supervisors as autocratic compromisers, again lacking developer and executive behaviors. This is probably why their work engagement was the lowest among all clusters, the same as work satisfaction, trust in the manager, and acceptance of the manager, while their willingness to change job was the highest among all four investigated groups. Finally, those from cluster 3 (*n* = 62) retrospectively assessed their sense of security before the pandemic as very high—as high as in clusters 1 and 2—but experienced a serious drop in subjective security, such that they were similar in this respect to participants from cluster 4. Although they declared relatively high levels of work engagement and work satisfaction, they did not seem to trust their manager, and they declared a low level of acceptance of their supervisor and a relatively high turnover intention. They perceived their managers as compromisers and, to a lesser extent, as autocrats and deserters, which means that they believed that they could not make confident decisions and concentrated on employees who were helpful for their career or well-being, while they were ready to sacrifice those who were more competent but also more demanding (Reddin, 1990). Yet, they presumed that their supervisor lacked developer and executive styles. The only work-related evaluation that was relatively high in this cluster was atmosphere satisfaction, which possibly means that although they could rely on their supervisor, they could rely on their coworkers.

## Discussion

Work environment, being one of the most important areas of self-expression for adults, can modulate the responses of employees to global crises. The quickly developing pandemic caused by a completely new and still incurable coronavirus demanded immediate reactions tailored to unique socioeconomic circumstances, cultural norms, and work-style preferences and requires knowledge, skills, and adequate tools to be used by leaders, as well providing up-to-date information about the virus. However, at the beginning of the crisis, it was extremely difficult—both for employers and employees—to implement effective countermeasures. For example, Kandel et al. (2020) found that 28% of a total of 182 examined countries had insufficient preventive capacities for dealing with the COVID-19 outbreak, while for 33%, these capacities were very low. Some countries did not manage to implement adequate preventive measures or provide clear information to the public, which in turn resulted in panic (Wenliang and Ghebreyesys, 2020). These difficulties were unavoidable at the beginning of the crisis, but we still need to develop effective coping strategies for now and for the future. Pacheco et al. (2020) indicated that during the COVID-19 pandemic, employees who worked in a workplace “prepared for disaster” exhibited subjective workplace security and higher well-being. In this work, we presumed that successful leaders may decrease both objective danger and the subjective sense thereof, protecting the physical and mental health and the economic condition of employees. During the COVID-19 pandemic, the managers can greatly influence organizational attitudes, such as organizational trust (Guzzo et al., 2021), and hence become one of the tools for global protection of citizens.

In this research study, we analyzed a snapshot of the situation at the beginning of the COVID-19 pandemic. The data collection started on March 13, 2020, when 132,758 people globally had been infected and 4,695 had died. In Poland, only 49 cases and 5 deaths had been confirmed at the beginning of the data collection process (WHO, 2020), so that the COVID-19 pandemic was more a subjective than an objective threat. We investigated subjective sense of workplace security at this time and retrospectively assessed the subjective sense of workplace security before the pandemic. Using the cluster analysis, we were able to connect it to work-related attitudes, such as work engagement, satisfaction and trust in manager, turnover intention, and to the perceived leadership styles ascribed to the managers of the participants. We found that the compilation of highly constructive and lowly ineffective management styles observed in supervisors was linked to a greater work engagement, satisfaction, manager assessment, lower turnover intention, and the highest levels of subjective workplace security. However, similar but weaker effects may exist among those who are led by managers focused on bureaucratic procedures: scheduled and formally controlled actions may also produce a sense of control over uncertain circumstance and hence sustain a relative sense of security, but they do not provide employees with the highest level of work satisfaction. Finally, participants who were supervised by compromising autocrats expressed the highest drop in security at the beginning of the crisis, but whether their level of work satisfaction was low seems to be linked with atmosphere satisfaction and trust in manager.

Our results support the concept of situational management, indicating that whether a specific management style is effective or not depends on external conditions, transient needs, and the situational context. The bureaucratic style—sometimes perceived as negative—can indeed have a positive effect on the subjective sense of security in critical situations because of its strong concentration on formal rules and because of a constant control. In unpredictable circumstances, the employees may show a strong preference for strict hierarchies (Friesen et al., 2014), rules, and order (Kay et al., 2009), and when led by bureaucratic supervisors, they may feel safer than others—although they would be less comfortable with such managers under standard conditions. Finally, autocratic and compromising styles are connected with the lowest sense of workplace security, a lack of trust in the manager, and the most negative work attitudes—they potentially result in the lowest employee well-being. However, a good work atmosphere might work as a protective buffer against these negative outcomes. Indeed, job demands–resources model (Demerouti et al., 2001) posits that support from colleagues can serve as a job resource that reduces the negative psychological consequences of job demands, such as work overload, role conflict, role stress, shift work, time pressures, or emotionally demanding work (Bakker and Demerouti, 2017). Job resources, such as a good work atmosphere, create opportunities for the development and personal growth and initiate motivational processes that may lead to a higher work engagement (Bakker and Demerouti, 2017).

We believed that our findings correspond to the observation that combining concentration on relations with executive determination may play a main role in the crisis management. After the SARS epidemic, 17 years ago, Smith (2006) observed disproportions between scales of health risk and economic costs and raised concerns that outbreaks of more serious diseases could cause catastrophic impacts on the global economy. He concluded that “serious infectious disease outbreaks need to be identified and dealt with quickly. Adverse health and economic effects can be reduced by early detection and response” (p. 3120). He especially stressed the importance of individual adequate risk perception, risk communication, and tailored management—factors that can be easily implemented as crisis management strategies in different types of work environments—as critically important predictors of constructive pandemic responses. The actions that seem to be the most important tools for coping with a pandemic in the workplace include not only a reducing contact with the virus (e.g., providing personal protective equipment and allowing working from home) but also providing informational support (e.g., open communication, including employees in the decision-making process) (Smith, 2006). Tseng et al. (2005), also drawing lessons from the SARS epidemic, identified five determinants of successful coping with global infections, namely, appropriate timing for crisis management (the earlier the better), careful decision-making (including employees in the process), thorough implementation, effective communication, and trust between management and employees. Highly stressful circumstances may increase the frequency of conflicts between employees and worsen the atmosphere, but implementing an executive and developer leadership style might be helpful in coping with the effects of a crisis in the workplace (Limbare, 2012). An efficient crisis management has been also found to correlate with charismatic leadership and, even more strongly, with transformational leadership (Sorsa, 2015). This may suggest that cognitive and behavioral flexibility is an essential managerial skill for coping with an unknown danger.

## Limitations and Future Directions

One of the obvious limitations of our study is its cross-sectional design, making it impossible to derive causal statements about the relation between our variables of interest. Furthermore, our sample was relatively small and not representative even of the Polish population, which limits the ability to generalize our results to a wider range of employees representing diverse professions, work environments, relations between employees and managers, etc. Although it could be worthwhile to replicate our results in a bigger sample, preferably including employees from diverse countries and a more systematic way of recruiting them to participate in the study to confirm the robustness of the effects, it would not be possible to do so. Since the beginning of 2020, circumstances change so fast that the situations of employees might be completely different each month, and we could not replicate data collection for the very early stage of pandemic. Future research should collect the longitudinal data to better understand the causality of the effects we investigated. Additionally, our study was based on self-report questionnaires. Even though the perceptions of employees of their managers are an important source of information, these perceptions might not reflect the objective work reality, as they might be affected by many additional factors, like general work satisfaction, personal likes and dislikes, recently received rewards and benefits, length of time working for the company or position, and the characteristics of employees, such as psychological capital^1^. Another limitation comes from the methods we applied to measure our variables of interest. Although the theory of Reddin (1967, 1990) is classic and widely used, the nature of the questionnaire gives limited possibilities for the analysis since the number of points always adds up to 8 for all the dimensions and because the reliability of measurement operationalized as internal consistency might be low. However, such a method gives clear information about the dominant leadership style perceived in the supervisor, and it is very easy for the respondents to comprehend. Finally, our other variables were measured with single items, and although they seem straightforward, their actual validity and reliability are unknown. Hence, there is a need to collect additional data, using well-established psychological methods measuring sense of security, work satisfaction, work attitudes, as well as supervisor management style, most preferably collecting the data from various employees working under the same manager and analyzing the data in a nested design.

## Final Conclusion

Almost 15 years ago, Smith (2006) stated that the 2001 SARS pandemic was the most striking example of a global socioeconomic solution to a widespread threat that we have observed in modern times. This statement now seems extremely outdated. We hope that our study provides some information about the importance of leadership skills and styles for managing in the time of a pandemic outbreak. It is also a good starting point for further research on the role of human resources management as well as the importance of work-related factors in successful leadership during global health crises, which might recur in the near and/or distant future. The study encourages further research involving representative groups of participants from various countries in Europe and the world, also taking into account the importance of national strategies for counteracting the spread of the coronavirus and reducing the negative effects of the pandemic.

## Data Availability Statement

The original contributions presented in the study are included in the article/Supplementary Material, further inquiries can be directed to the corresponding authors.

## Ethics Statement

Ethical review and approval was not required for the study on human participants in accordance with the local legislation and institutional requirements. The patients/participants provided their written informed consent to participate in this study.

## Author Contributions

AW was the principal coordinator of the project, was involved in the methodology design, managed the data collection process, wrote the manuscript, and performed the entire statistical analysis and interpretation of results. ET was involved in the data collecting, and writing the manuscript. KS was involved in the study design and data collecting process. AG supervised statistical analyzes and wrote the manuscript. All the authors had a significant and substantive contribution to the publication.

## Conflict of Interest

The authors declare that the research was conducted in the absence of any commercial or financial relationships that could be construed as a potential conflict of interest.

## Publisher's Note

All claims expressed in this article are solely those of the authors and do not necessarily represent those of their affiliated organizations, or those of the publisher, the editors and the reviewers. Any product that may be evaluated in this article, or claim that may be made by its manufacturer, is not guaranteed or endorsed by the publisher.

## Footnotes

^1^Penza A., and Gasiorowska, A. (under review). Linking work events with work engagement: Mediating role of emotions and moderating role of psychological capital. *J. Pers. Psychol*.
